# Research on the Relationship Between High-Commitment Work Systems and Employees’ Unethical Pro-organizational Behavior: The Moderating Role of Balanced Reciprocity Beliefs

**DOI:** 10.3389/fpsyg.2021.776904

**Published:** 2021-12-13

**Authors:** Min Zhang, Lijing Zhao, Zhihong Chen

**Affiliations:** ^1^Economics and Management School, Nantong University, Nantong, China; ^2^Business School, Nanjing University, Nanjing, China; ^3^Institute for International Students, Nanjing University, Nanjing, China

**Keywords:** high-commitment work systems, relational psychological contract, unethical pro-organizational behavior, balanced reciprocity, social exchange

## Abstract

Based on the social exchange theory, this paper explores the indirect impact of high-commitment work systems on employees’ unethical pro-organizational behavior. Through the analysis of multisource data from 139 companies (including 139 human resource managers and 966 employees), a multilevel structuring equation model is used to verify the study’s hypotheses. The research results show the following findings: (1) High-commitment work systems are significantly positively related to employees’ unethical pro-organizational behavior. (2) High-commitment work systems have indirect effects on the employees’ unethical pro-organizational behavior through the relational psychological contract. The relational psychological contract plays a mediating role in this process. (3) Employees’ balanced reciprocity beliefs significantly enhance the positive effect of relational psychological contracts on employees’ unethical pro-organizational behavior. It can also positively moderate the mediating effect of high-commitment work systems that affect employees’ unethical pro-organizational behavior *via* relational psychological contract.

## Introduction

In an economic environment of increasing competition, uncertainty, and complexity, managers try to stimulate employees’ pro-organizational behavior through various methods to obtain a sustainable competitive advantage. However, to achieve this goal, employees may look for shortcuts that violate the moral standards at for-profit organizations, ranging from tampering with financial data to withholding negative information from the public. Other unethical workplace behaviors were revealed in the “Enron event,” “Sanlu melamine incident,” and other corporate scandals, which become widespread (Xu and Lv, 2018). Many ethical misdeeds are destructive (such as damaging equipment) or purely self-serving (such as fraudulent reporting). Still, such misconducts stem from a desire to benefit the organization. To distinguish this from general unethical behavior, Umphress et al. (2010) put forward the concept of “unethical pro-organizational behavior,” which they defined as an act aimed at promoting the effective operation of the organization or its members (such as leaders) and violating the core social values, ethics, laws or standards of proper conduct (Umphress and Bingham, 2011). It is precise because unethical pro-organizational behaviors violate widely accepted ethical norms in society, which may eventually lead to harmful consequences (Vadera and Pratt, 2013), such as fines, corporate reputation tarnished, and damage to the interests of external stakeholders and even the whole society (Umphress and Bingham, 2011). Therefore, unethical pro-organizational behavior quickly became the focus of scholars and managers, who seek to identify more factors affecting unethical pro-organizational behavior in order to reduce or avoid such behaviors among employees.

Previous studies have studied the antecedents of unethical pro-organizational behavior from the perspectives of individual characteristics (such as psychological rights, moral identity, moral disengagement, the high performance expectation, and the high performance pressure; Wu et al., 2016a; Chen and Liang, 2017; Zhao and Zhou, 2017; Lee et al., 2019), leadership style and behavior (such as transformational leadership, and moral leadership; Miao et al., 2013; Effelsberg et al., 2014; Zhang et al., 2018; Wang and Li, 2019), and colleague behavior (Thau et al., 2015). However, it is worth noting that employees often engage in unethical pro-organizational behavior for the sake of organization’s short-term interests. What organizational situations encourage employees to show more willingness to participate in unethical pro-organizational behavior? According to the recent research, organizational context that predicts employees’ unethical pro-organizational behavior has not received sufficient attention (Xu and Lv, 2018). Scholars pay more attention to the relationship between organizational factors and employees’ unethical pro-organizational behavior, such as organizational identity, organizational emotional commitment, and organizational culture (Umphress et al., 2010; Alexandra, 2012).

In an organization, strategic human resource management aims to convey and strengthen the consistency of the relationship between employees and the organization to employees, and provides clear guidance on how the organization trains and supports employees and what the company expects from employees in return (Lepak and Snell, 1999; Bowen and Ostroff, 2004). High-commitment work systems, which are committed to the common development of employees and the organization, have the closest contact with employees (Chang et al., 2014). Under the guidance of the high investment concept, high-commitment work systems promote the development of employees’ skills and abilities through high investment measures, and help employees establish attachment and emotional commitment to the organization (Arthur, 1994; Lepak and Snell, 1999). By investing resources in its employees, high-commitment work systems try to establish trust, mutual exchange, and long-term relationships with employees (Walton, 1985; Whitener, 2001; Hauff et al., 2014), and when employees feel the “valuable things” provided by the organization, they are willing to offer “valuable things” in return, which may show more pro-organizational behavior (Ali et al., 2020). For example, for the organization’s benefit, even in the face of violating existing rules and moral standards of the organization, employees will choose to engage in behaviors beneficial to the organization, that is the occurrence of unethical pro-organizational behavior (Kehoe and Wright, 2013; Kehoe and Collins, 2017). Further cycles of mutually beneficial exchanges may occur when both parties in a relationship gain benefits (Mostafa et al., 2019), in keeping with the aphorism, “The grace of dripping water should be repaid by the spring.” When employees feel supported and cared for by the organization, will they choose to engage in unethical behaviors that benefit the organization’s interests because of their intense gratitude to the organization and their willingness to reward the organization? This research’s primary goal is to explore the relationship between high-commitment work systems and employees’ unethical pro-organizational behavior.

To clarify the mechanism of the high-commitment work systems on an employee’s unethical pro-organizational behavior, this research intends to further explore the mediation mechanism of the high-commitment work systems affecting an employee’s unethical pro-organizational behavior. The social exchange theory asserts that if one party pays for the other party and fulfills the other party’s expectations accordingly, then in response, the beneficiary will show positive behaviors and attitudes (Blau, 1964; Peyton et al., 2019). There is a long-term, unspecified obligation between the two parties in this process, which provides a theoretical framework for explaining how the organization’s human resource management practices affect employees’ work performance (Li et al., 2017). High-commitment work systems that highlight the “promise maximizer” provide employees with resources, support, and participation (Arthur, 1994), allowing employees to gain positive work experience, establish a sense of trust in and commitment to the organization, and form a long-term psychological contract. The establishment of a relational psychological contract based on mutual trust and long-term commitment, beyond the written form of agreement (Morrison and Robinson, 1997), will directly affect individuals’ attitudes and behaviors at work (He et al., 2019). Hence, taking positive reciprocity as the criterion of action (Gouldner, 1960), for the sake of repaying the organization, employees abide by the relational psychological contract by engaging in behaviors beneficial to the development of the organization, which sometimes may damage the interests of external stakeholders to a certain extent. Therefore, the relational psychological contract may mediate between the high-commitment work system and the employees’ unethical pro-organizational behavior.

Moreover, considering that employees’ unethical pro-organizational behavior occurs in the context of two-way interaction between employees and the organization, this research also incorporates employees’ perception of the organization’s equality and reciprocity into this research framework based on the social exchange theoretical framework. Compared to employees with a low level of balanced reciprocity beliefs, employees with a stronger belief in balanced reciprocity are more sensitive to the exchange of rights and interests (Huseman et al., 1987; Smith, 2003). Having perceived the material resources and psychological support given by the organization, they tend to immediately repay the organization with the same effective reward (Sparrowe and Liden, 1997). After forming a relational psychological contract with the organization, affected by the strong belief in equality and reciprocity, they are more likely to deliberately take actions that are beneficial to the organization but violate social laws or ethics to meet the organization’s requirements and realize the return to the organization as soon as possible in the short term. Therefore, this study believes that the stronger the employee’s balanced reciprocity beliefs is, the more obvious the effect of relational psychological contract on the employees’ unethical pro-organizational behavior may be.

In summary, based on social exchange theory, a cross-level model of high-commitment work systems affecting employees’ unethical pro-organizational behavior is deduced, so as to better understand the situational factors influencing employees’ choice of unethical pro-organizational behavior to repay the organization. At the same time, employees’ perception of organizational reciprocity will also play a role in high-commitment work systems’ effectiveness. Therefore, this research introduces the concept of balanced reciprocity beliefs and discusses whether balanced reciprocity beliefs determine the conditions for high-commitment work systems to affect the employees’ unethical pro-organizational behavior through relational psychological contract.

## Theoretical Basis and Research Hypothesis

### High-Commitment Work System and Employees’ Unethical Pro-organizational Behavior

In enterprises, when the organization implements the high-commitment work system, strictly selects employees in accordance with the requirements of organizational culture and norms, and then shapes and guides employees to develop specific skills and abilities through systematic training activities, employee participation in decision-making, reasonable salary and incentive (Bowen and Ostroff, 2004), and further promotes employees’ professional development and growth (Arthur, 1994; Chang et al., 2014). Based on the principle of reciprocity, as the return of high-level investment by the organization, employees often have strong pro-organizational will and motivation, which urges them to repay the organization and maintain the positive social exchange relationship between the two parties with pro-organizational behavior (Kalshoven et al., 2016). However, to maintain the high-quality social exchange relationship generated by the high level of investment in employees, employees will greatly reduce the possibility of behaviors that harm the interests of the organization, and even lower their own moral threshold and engage in behaviors that violate moral standards for the short-term interests of the organization (Wilks, 2011). In addition, employees with strong pro-organization motivation tend to ignore ethical and moral constraints to realize the return to the organization, and rationalize the attribution for their unethical behaviors (Wang et al., 2018), thus reducing the sense of cognitive dissonance brought by unethical pro-organizational behavior. Therefore, based on social exchange theory, this study argues that employees are more likely to engage in unethical pro-organizational behavior in return for the organization’s investment in them, when the organization effectively implements high-commitment work systems. Accordingly, the following hypothesis is proposed as:

H1: High-commitment work systems have a significant positive effect on employees’ unethical pro-organizational behavior.

### Mediating Effect of Relational Psychological Contract

Rousseau (1989) believed that psychological contract is the belief that there are mutual obligations between employees and organizations, while relational psychological contract is a long-term employment relationship established on the basis of mutual trust and loyalty. It is characterized by universality and openness, and there are no explicit obligations or performance requirements between both parties (MacNeil, 1985; Robinson and Rousseau, 1994; Raja et al., 2004). When a relational psychological contract is established between an organization and its employees, a stable relationship of mutual commitment exists between both parties (Rousseau, 1990, 1995; Janssens et al., 2003). It is worth noting that whether a relational psychological contract can be successfully established between an organization and employees is affected by the enterprise’s human resource management practice (Marks, 2001). Based on this, this study infers that as a human resource management strategy with a high level of investment, the high-commitment work systems emphasize high investment in employees and long-term participation of employees, which are conducive to shaping positive emotions and relationships between employees and the organization and inducing the formation of relational psychological contracts between both parties.

Specifically, first, in the strict employee screening stage, the organization selects employees who meet the enterprise’s development goals. When the organization and employees have a clear shared vision, it is easier for both parties to establish long-term emotional connection and relational psychological contract (Sun et al., 2007). Moreover, at the beginning of the employment relationship, organizations can lay the foundation for the formation of relational psychological contract by conveying the terms and conditions of the employment relationship, demonstrating the management philosophy behind human resource management practice (Suazo et al., 2009).

Secondly, high-commitment work systems involve a wide range of training activities to help new employees understand their roles, quickly obtain work-related knowledge and skills. Also such systems provide employees with the most direct perception of the organization’s investment and possible long-term employment opportunities, which can improve employees’ willingness to keep a good relationship with the organization. Thus, relational psychological contract between employees and organizations can be formed (Latorre et al., 2016; He et al., 2021).

Thirdly, the organization attaches importance to teamwork and emphasizes team performance appraisal, which is conducive to creating an excellent working atmosphere. The cooperation among team members will continuously stimulate the employees’ work potential, and the positive feedback from team leaders during the team performance evaluation process may indicate the establishment of different types of psychological contracts (Suazo et al., 2009). This belief makes employees more willing to develop a long-term oriented social exchange relationship based on social emotion with the organization, so as to establish a relational psychological contract (Rousseau and Schalk, 2000).

Fourthly, the measures, such as employee participation, higher salary level, and superior welfare, emphasized by the high-commitment work systems make employees realize that the organization values employees’ contributions enhancing their sense of trust and dependence on the organization, and thus generating long-term emotional resources and finally forming high-quality social exchange relationships (Arthur, 1994).

According to social exchange theory, employees with relational psychological contracts try to strengthen their relationship with the organization by adopting long-term commitments and engaging in more behaviors and obligations beyond their roles’ requirements (Choi et al., 2014), even though these behaviors may damage the organization’s long-term interests. Specifically, on the one hand, in the relational psychological contract, employees have an accurate perception of the responsibilities undertaken by the organization (Wu et al., 2006) and a strong desire to maintain a long-term employment relationship with the organization (El Akremi et al., 2010; Liu et al., 2013; Jawahar et al., 2018). Therefore, they are more likely to perceive that the benefits brought by unethical behaviors outweigh the costs, so they break away from the restrictions and constraints of morality (Becker, 1968; Lewicki, 1983) and regard unethical pro-organizational behavior as an “effective way” to maintain relationships (Cropanzano and Mitchell, 2005). On the other hand, a relational psychological contract means that the organization provides stable wages and long-term employment and supports the welfare and interests of employees and their families (Jepsen and Rodwell, 2012), which will significantly improve employees’ enthusiasm for repaying the organization. With the care and support of the organization, it is easy for employees to mistakenly think that the pro-organization unethical behavior is recognized by the organization. Although unethical pro-organizational behaviors violate ethical standards and even damage external stakeholders’ interests and the organization’s long-term development, such behaviors express their devotion and recompense to the organization. Moreover, these behaviors will not bring negative consequences and costs in a short term, like direct or indirect punishment and damage to its reputation or moral identity (Wang et al., 2018), and even have some benefits to the organization. Accordingly, the following hypothesis is proposed as:

H2: The relational psychological contract plays a mediating role in the impact of high-commitment work systems on employees’ unethical pro-organizational behavior.

### Moderating Effects of Balanced Reciprocity Beliefs

Sahlins (1972) proposed different types of reciprocity by examining different dimensions of reciprocity, namely, equivalence of income, timeliness of income and the degree, and nature of interests of both parties in the transaction, which are generalized reciprocity, balanced reciprocity, and negative reciprocity. As a positive reciprocal norm with altruism tendency, generalized norm aims to establish a long-term exchange relationship. In the process of exchange, all parties show a spirit of benevolence and self-sacrifice. On the contrary, negative reciprocity is a highly self-interest behavior whose purpose is to safeguard and maximize their own interests as much as possible. Sometimes, in order to achieve this purpose, it will even damage the interests of others. Balanced reciprocity is between generalized reciprocity and negative reciprocity, which emphasizes a high degree of reciprocity, the immediacy of return, and the common interests of both parties. This form of reciprocity comes from the strict and fine accounting of inputs and results by both sides of the exchange on the basis of trust. It emphasizes the criterion of “economic exchange,” which directly reflects people’s views on the exchange relationship (Sahlins, 1972; Wu et al., 2016b). On the whole, people’s calculation of time and value of extensive reciprocity is not only limited by the gift, but also depends on each other’s needs and appropriate time, which means that rewards may arrive quickly, but they may never materialize. In comparison, the interaction between both parties of balanced reciprocity has clearer economic and social purposes, and in the process of exchange, the social relations between actors change along with the changes of economic relations. Therefore, we infer that balanced reciprocity is more likely to affect the relationship between relational psychological contract and employee unethical pro-organizational behaviors.

Social exchange theory further states that individuals are concerned with balancing giving and receiving in social exchange. In other words, differences in the degree of individual recognition of “reciprocity” will affect the quality of social exchange relationships and influence the behaviors between employees and organizations (Cropanzano and Mitchell, 2005). Individuals with a strong belief in balanced reciprocity are committed to the exchange of equal resources. In other words, when employees establish a close relational psychological contract with the organization, they tend to repay the organization with something of equal value within a short period, devoting more to the work and the enterprise, and willing to contribute to the realization of the organizational performance of the enterprise based on the principle of giving back to the organization, and paying more attention to how to quickly benefit the organization in a short term, so as to increase the possibility of unethical pro-organization behavior. Simultaneously, unethical pro-organization behaviors can also reduce the sense of guilt caused by failure to provide feedback to some extent (Bredewold et al., 2016). On the contrary, if the individual’s belief in balanced reciprocity is low. It means that employees believe that after contributing to the organization, the organization ignores or neglects employees’ efforts, which will lead to the rupture of the relational psychological contract established between employees and the organization, so employees will stop paying and investing in their work, and will not violate moral laws and regulations in order to return to the organization in a short term and create “immediate benefits” for the organization, thus reducing their willingness to engage in unethical pro-organizational behavior. In this case, the influence of relational psychological contract on employees’ unethical pro-organizational behavior is weakened. Accordingly, the following hypothesis is proposed as:

H3: Employees’ balanced reciprocity beliefs moderate the relationship between relational psychological contract and their unethical pro-organizational behavior, that is, the stronger the employee’s belief in balanced reciprocity is, the more obvious the relationship between relational psychological contract and unethical pro-organizational behavior is.

The relationships revealed by hypothesis 2 and hypothesis 3 can be further understood as a moderated mediation model. Specifically, the relational psychological contract mediates high-commitment work systems’ influence on employees’ unethical pro-organizational behavior, but employees’ belief in balanced reciprocity also moderates this mediating effect. When employees believe that an organization has a high level of balanced reciprocity beliefs, the high-commitment work systems will positively impact the relational psychological contract. To repay the organization, establishing the relational psychological contract will further promote employees to make more unethical pro-organizational behaviors. Conversely, when employees’ belief in balanced reciprocity is weak, the relationship between relational psychological contract, high-commitment work systems, and employees’ unethical pro-organizational behaviors will be more fragile. Thus, the mediating effect of the relational psychological contract will be further weakened. Accordingly, the following hypothesis is proposed in this study:

H4: Employees’ balanced reciprocity beliefs moderate the mediating effect of the relational psychological contract between high-commitment work systems and employees’ unethical pro-organization behaviors. With the enhancement of employees’ balanced reciprocity beliefs, the mediating effect of relational psychological contract becomes stronger, otherwise, the mediation effect becomes weaker.

## Research Methods

### Samples and Procedures

This study’s data are collected from enterprises in Jiangsu, Anhui, Guangdong, Sichuan, Beijing, and other places. The types of enterprises include manufacturing, service industry, high-tech enterprises, and others. After obtaining consent from each enterprise’s human resources department manager, the researcher explained the survey’s purpose, process, and confidentiality. By mailing the paper questionnaire, each enterprise’s human resources department manager organized randomly selected employees to fill in the questionnaire and then send back the completed questionnaire.

To avoid the effect of homologous variance, this research collected data from multiple sources. The enterprise’s human resource department managers completed the questionnaire on the high-commitment work systems and the demographic variables at the enterprise level. Each enterprise’s employees completed the questionnaire of the relational psychological contract, balances reciprocity, unethical pro-organizational behavior, and the demographic variables at the individual level. A total of 150 enterprises were contacted before the survey, and 150 questionnaires for human resource department managers and 1,200 questionnaires for employees were delivered. After eliminating invalid questionnaires, 139 valid questionnaires were obtained of human resource department managers and 966 of employees, with effective recovery rates of 92.67 and 80.5%, respectively. Table 1 shows the sample characteristics of this research.

**Table 1:** Characteristics of samples.

Characteristics of Employees				Characteristics of Organizations			
Gender (%)		Education (%)		Types (%)		Numbers of employees (%)	
Male	55%	Middle school or below	10.8%	Stated-owned	47.5%	Less than 50	7.2%
Female	45%	Junior college	29.4%	Foreign	25.9%	50 ~ 100	10.1%
Age (%)		Bachelor degree	55.7%	Private	17.3%	101 ~ 500	41%
Less than 25	16.3%	Master degree or above	4.1%	Others	9.4%	501 ~ 1,000	22.3%
26 ~ 30	36%	Tenure by Year (%)		Organization age (%)		1,001 ~ 2000	5%
31 ~ 35	25.4%	Less than 1	17.6%	Less than 1	19.4%	More than 2001	14.4%
36 ~ 40	9.6%	1 ~ 3	30.6%	1 ~ 2	39.6%		
More than 41	12.7%	3 ~ 5	22.8%	2 ~ 4	23.7%		
		More than 5	29%	More than 4	17.3%		

### Measurement of Variables

The scales used in this study are all quoted from papers published in top journals abroad and translated into Chinese scales according to the procedure of translation and retranslation (Brislin, 1970). Likert seven points were used for all the scales involved, from “1-totally disagree” to “7-totally agree.”

### High-Commitment Work Systems

A scale of 10 items compiled by Xiao and Tsui (2007) was selected (Xiao and Tsui, 2007). For items, such as “Our company emphasizes open communication and wide information sharing, “the scale’s internal consistency coefficient is 0.900.

### Relational Psychological Contract

A scale of 5 items compiled by Hui et al. (2004) was selected (Hui et al., 2004). For items, such as “Our company is concerned for my long-term well-being,” the scale’s internal consistency coefficient is 0.914.

### Balanced Reciprocity Beliefs

The scale of 6 items compiled by Wu et al. (2006) was selected (Wu et al., 2016b). For items including “My organization takes care of the organization’s interests as much as my interest,” the scale’s internal consistency coefficient is 0.891.

### Employee Unethical Pro-organizational Behavior

A scale of 6 items compiled by Umphress et al. (2010) was selected (Umphress et al., 2010). One example includes “If my organization needed me to, I would withhold issuing a refund to a customer or client accidentally overcharged.” The internal consistency coefficient of this scale is 0.880.

### Control Variables

Previous studies believe that variables at the individual level (such as gender, age, education level, and tenure) and organizational level (such as enterprise type, enterprise size, and enterprise establishment years) may affect the occurrence of employees’ unethical pro-organizational behavior, and most empirical studies on unethical pro-organizational behavior, especially, the cross-level structural equation models constructed, take these variables as control variables (Umphress et al., 2010; Xu and Lv, 2018). Therefore, we also controlled for demographic variables at the individual and organizational levels. Specifically, human resource department managers fill in the control variables of the organization level, including enterprise type (1 “stated-owned,” 2 “foreign,” 3 “private,” and 4 “others”), numbers of employees (1 “less than 50,” 2 “50 ~ 100,” 3 “101 ~ 500,” 4 “501 ~ 1,000,” 5 “1,001 ~ 2000,” and 6 “more than 2001”), organization age (by year; 1 “less than 1,” 2 “1 ~ 2,” 3 “2 ~ 4,” and 4 “more than 4”); line staff fill in the demographic variables at the individual level, including gender (1 “male” and 2 “female”), age (1 “Less than 25,” 2 “26 ~ 30,” 3 “31 ~ 35,” 4 “36 ~ 40,” and 5 “more than 41”), education level (1 “middle school or below,” 2 “junior college,” 3 “bachelor degree,” and 4 “master degree or above”), and tenure (by year; 1 “less than 1,” 2 “1 ~ 3,” 3 “3 ~ 5,” and 4 “more than 5”).

## Data Analysis and Results

### Confirmatory Factor Analysis

This study used Mplus 7.40 software to distinguish the validity of the variables, the inspection through multiple levels of confirmatory factor analysis. The results showed that compared to other competitive models, four factors of measurement model have better fitting validity, *χ*^2^ = 614.461, *df* = 151, RMSEA = 0.056, CFI = 0.949, TLI = 0.938, SRMR (within-level) =0.037, SRMR (between-level) = 0.061. This study selected four variables with good validity, effectively representing four different research constructs.

### Descriptive Statistics and Correlation Analysis

Table 2 shows the mean value, standard deviation, and correlation of all study variables. Table 2 shows that relational psychological contract is positively correlated with employees’ unethical pro-organizational behavior (*r* = 0.574, *p* < 0.01), and equality and reciprocity are positively correlated with employees’ unethical pro-organizational behaviors (*r* = 0.177, *p* < 0.01), which preliminarily supports the research hypothesis.

**Table 2:** Descriptive statistics of variables.

	*M*	*SD*	1	2	3	4	5	6
Individual Level								
1. Gender	1.470	0.504						
2. Age	3.750	1.427	−0.067^*^					
3. Education	2.950	1.125	−0.008	−0.305^**^				
4. Tenure	54.230	55.189	−0.020	0.581^**^	−0.221^**^			
5. Relational Psychological Contract	4.065	1.073	−0.024	0.025	0.002	0.048		
6. Balanced reciprocity beliefs	4.947	1.125	0.018	0.022	−0.006	−0.057	0.120^**^	
7. Unethical Pro-Organizational Behavior	3.799	0.967	−0.021	−0.009	0.044	0.041	0.574^**^	0.177^**^
Organizational Level								
8. Types	1.885	1.008						
9. Numbers of Employees	3.504	1.380	−0.135					
10. Organization Age	28.806	23.652	−0.028	0.194^*^				
11. High-Commitment Work Systems	4.547	1.017	0.012	0.077	0.001			

** *p* < 0.01;

* *p* < 0.05.

*n*_employee_ = 966, *N*_organization_ = 139.

**Table 3:** Monte Carlo simulation tests the moderated mediating effect.

Dependent variables	Balanced reciprocity beliefs	Effect value	Standard error	Lower limit	Upper limit
Employees’ unethical pro-organizational behavior	High level	0.133^**^	0.047	0.043	0.230
	Low level	0.097^**^	0.034	0.032	0.167
	Difference	0.036^*^	0.021	0.003	0.085

** indicates *p* < 0.01;

* indicates *p* < 0.05.

Bootstrap times are 20, 000.

### Hypothesis Testing

**Figure 1:**
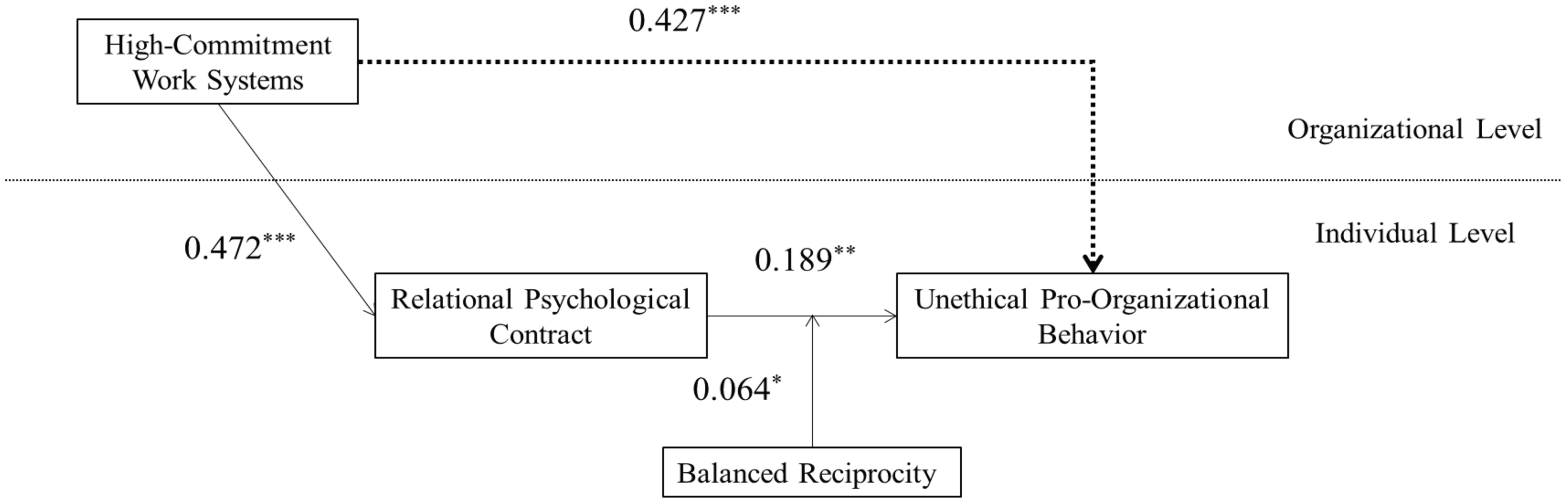
The final model with coefficients.

After controlling gender, age, education level, tenure on individual levels of, and enterprise type, enterprise size, and enterprise establishment years on the organizational level, this study establishes a multilevel moderated mediation model of high-commitment work systems’ unethical pro-organizational behavior. The result of path analysis is shown in Figure 1. As shown in Figure 1, high-commitment work systems significantly affect employee’s unethical pro-organizational behavior (*γ* = 0.427, *p* < 0.001). Hypothesis 1 is supported.

According to the multilevel model results in Figure 1, high-commitment work systems have a significant positive effect on relational psychological contracts (*γ* = 0.472, *p* < 0.001). The relational psychological contract also has a significant positive effect on unethical pro-organizational behavior (*γ* = 0.189, *p* < 0.01). Meanwhile, the direct effect of high-commitment work systems on employee’s unethical pro-organizational behavior remained significant (*γ* = 0.339, *p* < 0.001), suggesting that the relational psychological contract played a partial mediating role between high-commitment work systems and employees’ unethical pro-organizational behavior. Hypothesis 2 is supported. In addition, the results of 20,000 tests using the Monte Carlo method showed that a 95% confidence interval for the relational psychological contract’s mediating effect was (0.023, 0.162), and the interval did not include 0. This indicates that the relational psychological contract has a significant mediating effect between high-commitment work systems and employees’ unethical pro-organizational behavior. Hypothesis 2 is further verified.

**Figure 2:**
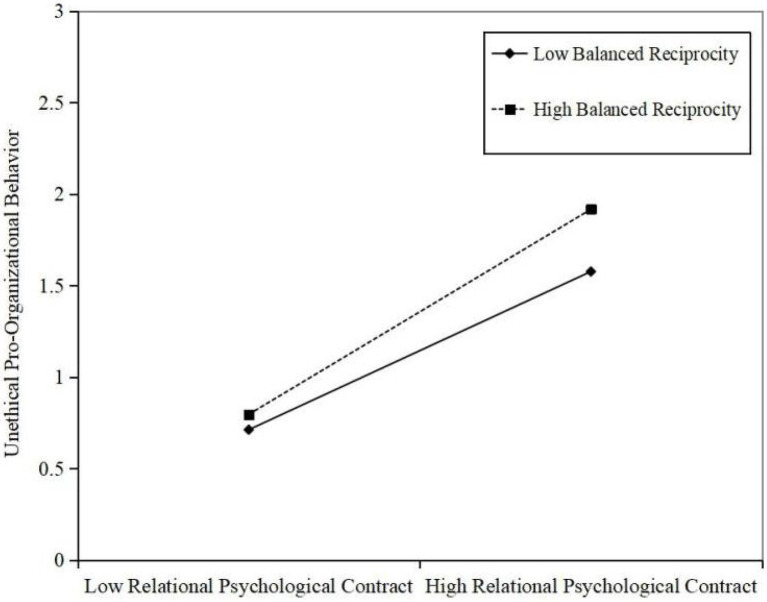
The moderating role of balanced reciprocity beliefs in the relationship between relational psychological contract and unethical pro-organizational behavior.

According to Figure 1, the interaction between relational psychological contract and balanced reciprocity beliefs has significant positive impact on employees’ unethical pro-organizational behavior (*γ* = 0.064, *p* < 0.05). It shows that balanced reciprocity beliefs have significant moderating effect between relational psychological contract and employees’ unethical pro-organizational behavior. Hypothesis 3 is supported. This study drew the moderating effect chart of balanced reciprocity beliefs more intuitively to reflect the moderating effect of balanced reciprocity beliefs between relational psychological contract and employees’ unethical pro-organizational behavior. As shown in Figure 2, when the level of balanced reciprocity beliefs is low, the relational psychological contract has a significant positive impact on employees’ unethical pro-organizational behavior (*γ* = 0.424, 95% LLCI = 0.345, 95% ULCI = 0.503). When the level of balanced reciprocity beliefs is high, the positive effect of relational psychological contract on employees’ unethical pro-organizational behavior is more significant (γ = 0.568, 95% LLCI = 0.489, 95% ULCI = 0.648), indicating that the moderating effect of balanced reciprocity beliefs has been verified. Therefore, hypothesis 3 is supported.

In this study, the Monte Carlo method was also used to test the moderated mediating effect. The R program was used to sample 20,000 times. Results are shown in Table 3. In the high level of balanced reciprocity beliefs, the indirect effect of high-commitment work systems on employees’ unethical pro-organizational behavior through relational psychological contract was 0.133, and the 95% confidence interval was (0.043, 0.230), which did not include 0. In the low level of balanced reciprocity beliefs, the indirect effect of high-commitment work systems on unethical pro-organizational behavior through relational psychological contract was 0.097; 95% confidence interval was (0.032, 0.167), and the interval did not include 0. Simultaneously, the difference between groups was 0.036, and the 95% confidence interval was (0.003, 0.085), reaching the significance level. This indicates that the mediating effect is different at different levels of balanced reciprocity beliefs; that is, the mediating effect of relational psychological contract is moderated by balanced reciprocity beliefs. Hypothesis 4 is supported.

## Discussion and Conclusion

This paper discusses the mechanism of high-commitment work systems’ influence on employees’ unethical pro-organizational behavior based on the social exchange theory. The results show that the high-commitment work system is significantly beneficial to establishing relational psychological contracts and directly promotes the occurrence of unethical pro-organizational behavior. The relational psychological contract mediates the influence of high-commitment work systems on employees’ unethical pro-organizational behavior. Employees’ balanced reciprocity beliefs positively moderate the relationship between relational psychological contract and employees’ unethical pro-organizational behavior, and positively moderate the relationship between high-commitment work systems and employees’ unethical pro-organizational behavior.

### Theoretical Contribution

This research has the following theoretical contributions. First, a review of the literature showed that most current studies on employees’ unethical pro-organizational behavior illustrate the occurrence process of such behaviors from the perspective of individual characteristics (e.g., organizational commitment and psychological rights; Umphress et al., 2010; Lee et al., 2019) or relationships (e.g., leadership style and colleague behavior; Miao et al., 2013; Effelsberg et al., 2014), but pay less attention to how the strategic HRM practices at the enterprise level influence employees’ unethical pro-organizational behavior. The occurrence of employees’ pro-organizational immoral behavior is often related to the specific management practice at the enterprise level, which is because the specific measures of strategic human resource management implemented by enterprises have the most direct and close contact with employees (Chang et al., 2014). The “effective solutions” implemented by the organization and managers may inhibit the occurrence of employees’ unethical behavior as expected by the organization but may also stimulate employees’ pro-organizational unethical behavior. Therefore, this study discusses that the implementation of high-commitment work systems that provide employees with many “benefits” is more likely to prompt employees to ignore ethical standards for the sake of the organization’s benefit, resulting in unethical behavior. This paper’s research conclusion effectively responds to the call put forward by scholars to study how to effectively avoid employees’ unethical pro-organizational behavior from the perspective of organizational context (Kalshoven et al., 2016), and to some extent, expands the research on the influencing factors that lead to the occurrence of employees’ unethical pro-organizational behavior.

Secondly, previous studies have emphasized more on the positive role of social exchange, while ignoring the possible negative consequences of social exchange. The emergence of employees’ unethical pro-organizational behavior is one of the evidences of the negative impact of social exchange (Kalshoven et al., 2016). Similarly, the “employee centered” high-commitment work systems aim to strengthen employees’ commitment to the organization, establish a good relational psychological contract between employees and the organization, promote the realization of organizational goals, and help employees achieve their own development. Consistent with the outcome variables expected by the organization, scholars pay more attention to and investigate the impact of high-commitment work systems on employees’ positive attitude and behavior (Farndale et al., 2011). In fact, based on the principle of social exchange, the implementation of high-commitment work systems may also promote employees to show behaviors beyond the expectations of the organization, including immoral behaviors that lose the long-term interests of the organization, which will have a fatal impact on the organization and employees. Therefore, this study’s conclusion is a useful supplement to the mechanism of how the previous organizational human resource management practices affect the employees’ unethical pro-organizational behavior, and at the same time, enriches the studies on the negative consequences brought by implementing high-commitment work systems.

Thirdly, based on the perspective of reciprocity principle of social exchange theory, this study explains the internal mechanism between high-commitment work system and employees’ pro-organizational unethical behavior, and finds that the high-commitment work system implemented by the organization promotes employees to take pro-organizational unethical behavior to repay the organization by establishing and maintaining employees’ relational psychological contract. Although in previous studies, scholars have used social exchange theory to explain the mediating mechanism of pro-organizational immoral behavior (Umphress et al., 2010), no research has tested the role of psychological contract during this process. Therefore, this study makes contribution by selecting the relational psychological contract that has received less attention in the past but has a long-term mutually beneficial relationship with enterprises as mediator, revealing the mechanism of the impact of high-commitment work system on employees’ pro-organizational unethical behavior, and retesting the explanation of social exchange theory for pro-organizational unethical behavior.

Finally, this study investigates the balanced reciprocity beliefs as a contingency factor, influencing the occurrence of unethical pro-organization behaviors. According to the social exchange theory, the individual’s perception of the degree of reciprocity with the organization will lead the individual to adopt different behaviors. Employees with a firm belief in balanced reciprocity will more likely show unethical pro-organizational behavior to maintain an excellent relational psychological contract with the organization. Therefore, this study incorporated the balanced reciprocity beliefs as an individual factor into the research framework of the influence of high-commitment work systems on employees’ unethical pro-organizational behavior and considered the joint effect by organizational context factors and personal factors on the occurrence of employees’ unethical pro-organizational behavior. This study’s conclusions contribute to a more comprehensive understanding of the boundary conditions of organizational HRM practices affecting unethical pro-organizational behavior.

### Practical Inspiration

This research also has specific management significance. First of all, managers should be vigilant against the occurrence of employees’ pro-organizational unethical behavior, and put forward relevant management suggestions. Although in the short term, employees’ pro-organizational unethical behavior may benefit the organization, several related events (e.g., “Enron event” and “Sanlu melamine include”) show that the occurrence of such behavior will not only affect the economic benefits of the organization, but also damage the reputation of the organization. Therefore, managers should try their best to make employees realize that the organization cannot tolerate the occurrence of unethical behavior and cannot make employees mistakenly believe that pro-organization unethical behavior is the default or advocacy of the organization. Employees should be encouraged to match the organizational culture and values of the enterprise’s long-term development for the sake of the long-term development of the organization.

Secondly, the study concludes that implementing high-commitment work systems may lead to employees’ unethical pro-organizational behavior, and thus provides suggestions for enterprises’ managers. Even the high-commitment work systems committed to the common development of employees and organizations may have a negative impact. Therefore, in the process of implementing the high-commitment work systems, attention should be paid to avoid or reduce the possibility of employees’ unethical pro-organizational behavior, such as selecting employees with high moral level in recruitment, inform employees of correct moral values and code of conduct during training, include moral evaluation standards in the evaluation, regular lectures, and thematic discussions on ethics (Xu and Lv, 2018). In short, in the recruitment, training and daily management of employees, enterprises should pay attention to cultivating employees’ awareness of social responsibility and guiding employees to work in a correct way.

Thirdly, with relational psychological contract as a “double-edged sword,” managers should pay attention to the benign social exchange relationship between employees and organizations, carry out human resource management practices conducive to the establishment of trust and long-term exchange relationship. Enterprises should be trustworthy and keep their promises, and maintain the psychological contract with employees by actively fulfilling their commitments, and furthermore guide the pro-organizational behavior that employees may make in order to repay the organization. At the same time, department leaders, heads of human resources management department, and other managers should still pay attention to the observation of employees’ behavior after the establishment of relational psychological contract, so as to reduce or avoid employees’ unethical behavior for legitimate reasons, such as meeting organizational expectations and returning to the organization.

Finally, managers should be aware of the positive and negative impact of employees’ belief in balanced reciprocity on their behavior. On the one hand, we should build an effective incentive mechanism of balance and mutual benefit, give timely incentives and feedback to employees’ work results, and create an organizational atmosphere of mutual benefit; On the other hand, a high level of belief in balanced reciprocity may also stimulate employees’ unethical pro-organizational behavior. Therefore, managers should carefully cultivate employees’ belief in equality and reciprocity, and emphasize employees’ moral and ethical awareness while establishing clear moral norms (Liu et al., 2020).

## Limitations and Future Research

This study still has the following limitations, which should be further improved in the follow-up study. Firstly, in terms of research design, this study mainly verifies the theoretical model through empirical research. The research results are based on cross-sectional data, that is, the data of main variables and control variables of this study are collected at the same time point. Therefore, future research can provide more convincing evidence for research hypotheses through vertical research. In addition, the human resources managers and employees of each research unit are invited to fill in the questionnaire. Although the data sources are different, they may still be affected by the common method deviation. Then, the follow-up research can consider improving the external validity of the data through experimental design or collecting objective data. For example, besides the traditional questionnaire measurement method, some scholars use scenario to measure employees’ unethical pro-organizational behavior (Wang et al., 2021), which is worthy of reference for future scholars in empirical research.

Secondly, this study is still an intermediary mechanism proposed under the framework of social exchange theory, which is similar to previous empirical studies on employees’ pro-organizational immoral behavior, but it needs to be further explored whether there are other possible mechanisms (such as cognitive imbalance, social comparison, and negative affect), to promote or inhibit employees to engage in unethical pro-organizational behavior (Chen et al., 2016; He et al., 2020), for the purpose of continuously expanding the research on the intermediary mechanism of employees’ unethical pro-organizational behavior. Also, this study mainly focuses on the moderator role of employees’ belief in balanced reciprocity in the generation of their pro-organizational immoral behavior. Follow-up research can further explore whether there are different contextual variables (such as organizational ethical atmosphere and organizational culture) or individual factors (such as values and power distance) influencing employees’ judgment on ethical standards, so as to stimulate or inhibit the possibility of unethical pro-organizational behavior (Graham et al., 2019; He et al., 2020).

Lastly, although this study controlled the relevant demographic variables from the individual and organizational levels, from the perspective of data analysis, the controlled variables did not have a substantive impact on the research results. Therefore, scholars can consider putting forward effective controlled variables on the basis of theoretical reasoning.

## Data Availability Statement

The raw data supporting the conclusions of this article will be made available by the authors, without undue reservation.

## Author Contributions

All authors listed have made a substantial, direct and intellectual contribution to the work, and approved it for publication.

## Funding

This research project was supported by National Natural Science Foundation of China (No. 71832007; 72072081); National Social Science Foundation of China (No. 20AGL020).

## Conflict of Interest

The authors declare that the research was conducted in the absence of any commercial or financial relationships that could be construed as a potential conflict of interest.

## Publisher’s Note

All claims expressed in this article are solely those of the authors and do not necessarily represent those of their affiliated organizations, or those of the publisher, the editors and the reviewers. Any product that may be evaluated in this article, or claim that may be made by its manufacturer, is not guaranteed or endorsed by the publisher.
